# Dynamics of Polymer Rings in Ring-Linear Blends by Neutron Spin Echo Spectroscopy

**DOI:** 10.1021/acsmacrolett.5c00507

**Published:** 2025-09-15

**Authors:** Margarita Kruteva, Jürgen Allgaier, Michael Monkenbusch, Peter Falus, Katerina Peponaki, Dimitris Vlassopoulos, Dieter Richter

**Affiliations:** † Jülich Center for Neutron Science, 28334 Forschungszentrum Jülich, 52428 Jülich, Germany; ‡ 56053 Institut Laue-Langevin (ILL), 71 rue des Martyrs, 38042 Grenoble, Cedex 9, France; § FORTH, 124215 Institute for Electronic Structure and Laser, Heraklion 71110, Greece; ∥ University of Crete, Department of Materials Science and Engineering, Heraklion 70013, Greece

## Abstract

We present a microscopic investigation of the polyethylene-oxide (PEO) ring dynamics in symmetric ring-linear blends with a molecular weight of 40 kg/mol over the full concentration range. Applying neutron spin echo (NSE) spectroscopy on samples containing a fraction of labeled rings, we observe the internal ring dynamics and its modifications as a function of ring volume fraction ϕ_R_. With increasing linear composition, a dynamic cross over from self-similar ring-like relaxation to local reptation-like dynamics is observed. At ϕ_R_ = 0.5, where the blend viscosity exhibits its maximum, the spectral shapes change from ring- to local reptation-type dynamics, even though the enacted constraints are weaker than those in the linear melt. For ϕ_R_ ≤ 0.35, the ring motion is completely enslaved by the linear host.

## Graphical abstract

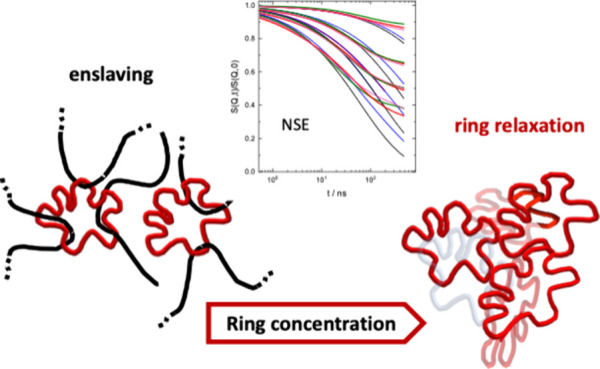

Blends of ring and linear polymers display emergent rheological properties: the blend viscosity at intermediate composition shows by factors larger values than that of the two neat components.^1–17^ So called architectural blends were investigated since Roovers pioneered the synthesis of large, well-defined ring polymers.^5^ Blending linear and ring polybutadienes corresponding to an entanglement number of *Z* = 15.3, Roovers found a maximum in the zero-shear viscosity η_0,max_ for a ring volume fraction of ϕ_R_ = 0.4.^4^ By large scale simulations with a bead spring model Halverson et al. reported that threading of rings by linear chains is the essential element determining the dynamics leading to “composite entanglements”.^7^ As Roovers experiments also simulations displayed a nonmonotonic dependence of the viscosity on the blend ratio with a maximum around ϕ_R_ = 0.5. Tsalikis et al. performed atomistic simulations on PEO systems of up to 10 kg/mol chain length finding that threading slows ring polymer relaxation by an order of magnitude or more.^10^ In blends the dynamics of rings was observed to be strongly affected but the linear chains were not influenced.^7^ By rheology and simulation Parisi et al. studied viscoelastic properties of ring-linear blends up to ϕ_R_ = 0.3.^18^ They found a linear increase of the zero-shear viscosity with increasing ring fraction by a factor of 2. The rationale of the modeling was that rings are trapped by threading of linear chains. Stress relaxation is possible only, if these threadings are released. Assuming a linear superposition of both moduli for the linear *G*_L_(*t*) and the ring component *G*_R_(*t*) they could explain quantitatively the experimental results. However, the linear superposition approach only works for low ring concentrations in the blend. Simulating symmetric ring-linear blends O’Connor et al.^15^ reported complex topological states with η_0,max_ around ϕ_R_ = 0.5: at small ϕ_R_ rings are interwoven or enslaved by the entanglement network of the linear chains, a phenomenon also observed by NSE experiments of Goossen et al.^8^ Replacing linear chains by rings dilutes linear–linear entanglements. For large ring fractions linear entanglements break down and are replaced by ring–ring and ring–linear threading. Addressing the dynamic modulus *G*(*t*) of ring melts, Kapnistos et al. discovered that linear chains in ring melts exhibit very strong influence on their rheological properties.^6^ The power law characteristics of *G*(*t*) is already suppressed at 1% linear, at 5% linear a rubbery plateau of the blend is found. Furthermore, ring diffusion in the blend decreases with increasing linear chain fraction. Above the ring–ring overlap concentration c* the diffusion is strongly affected. Detailed PFG-NMR investigations at high linear concentration revealed two diffusion processes: fast diffusion and a slow process controlled by constraint release.^16^ Finally, we note that for small ring sizes threading is not observed;^16^ also, a viscosity overshoot or a delay in the ring dynamics does not take place.^19–22^ Even though by now much is known about the rheological properties of ring-linear blends, and a significant number of simulations were reported, microscopic experiments on these systems are largely unavailable.

In this letter we present a microscopic experimental view on the dynamics of polymer rings in ring-linear blends. By neutron spin echo (NSE) spectroscopy we investigated the ring dynamics in such blends over the full concentration range. The following results stand out: (i) for ring volume fractions ϕ_R_ ≤ 0.35 the ring spectra, are identical to those for local reptation from the neat linear melt and demonstrate that the ring dynamics is completely enslaved by the linear chains; (ii) at ϕ_R_ = 0.5, where the blend viscosity exhibits its maximum, the ring spectra still show the features of local reptation but the effective entanglement distance is enlarged; seemingly here ring type threading together with the, however, weakened entanglement constraints, evoke a maximum effect on macroscopic dynamics such as viscosity; (iii) at higher ϕ_R_ the self-similar ring type spectra prevail, however, the lowest indexed relaxation modes, being the spatially most extended, are suppressed. (iv) The center of mass (com) diffusion displays a large subdiffusive regime with a crossover to Fickian diffusion that for ϕ_R_ = 0.75 extends to a crossover mean square displacement ⟨*r*_cross_^2^⟩ which equals to the linear end-to-end distance ⟨*R*_e_^2^⟩: on average, the ring has to move over the span of the ⟨*R*_e_^2^⟩ before it is freed from all initial constraints, a very descriptive feature.

Self-similar internal ring dynamics takes place in two steps: (i) At short times and distances the elementary loops of size *N*_e_ that build the ring conformation perform Rouse dynamics with mode relaxation times τ_*p*_ ∼ *p* ^–2^, where *p* is the mode number. (ii) For larger distances and times, the regime of loop relaxation follows. In terms of scaling theories, their mode spectrum has the form τ_*p*_ ∼ τ_2_ *p* ^–μ^, with μ = 2 + 1/*d*_f_ (*d*_f_: fractal ring dimension) and τ_2_ the relaxation time of the first ring mode (only even modes are allowed).^9^ Via a continuity condition at τ_e_, the Rouse time of the elementary loops, the two regimes are connected. With this the corresponding coherent dynamic structure factor for *S*_ring_(*Q*,*t*) becomes^13^

1 $$S_{\mathrm{ring}}\left(Q,t\right)=\frac{1}{N}\exp\left[-\frac{Q^{2}}{6}\left\langle r_{\mathrm{com}}^{2}\left(t\right)\right\rangle \right]\times \overset{N}{\underset{i,j}{\sum }}\exp\left[\frac{{\left(Ql_{\mathrm{seg}}\right)}^{2}}{6}{\left(\left|i-j\right|\frac{N-\left|i-j\right|}{N}\right)}^{2\nu_{i,j}}-B_{i,j}\left(t\right)\right]$$

where

$$\begin{array}{l} B_{i,j}\left(t\right)=\frac{2N^{2\nu_{i,j}}{\left(Ql_{\mathrm{seg}}\right)}^{2}}{3\pi^{2}}\overset{N}{\underset{p,\mathrm{even}}{\sum }}\frac{1}{p^{2}}f_{p}\left(p\right)\cos\left(\frac{p\pi \left|i-j\right|}{N}\right)\left[1-\exp\left(-t\Gamma_{p}\right)\right]\\ \Gamma_{p}=\frac{\left(1-T_{f}\left(p\right)\right)\pi^{2}Wl4}{N^{2}l_{\mathrm{seg}}^{4}}+T_{f}\left(p\right)w\pi^{2}{\left(\frac{p}{p_{\min}}\right)}^{\mu }{\left(\frac{p_{\min}}{N}\right)}^{2} \end{array}$$

with ⟨*r*_com_^2^(*t*)⟩ being the center of mass displacement, *l*_seg_ is the monomer length, and $w=\frac{Wl4}{l_{\mathrm{seg}}^{4}}$ is the monomer relaxation rate, and the scale independent “Rouse rate” is $Wl4=\frac{3k_{B}Tl_{\mathrm{seg}}^{2}}{\zeta }$ , with *k*_B_ the Boltzmann constant, *T* the temperature, and ζ the monomeric friction coefficient. In terms of a Rouse mode analysis, the factor *f*_*p*_ allows to modify the contribution of the Rouse mode *p* to the chain relaxation; in general, for the *p*, dependence of *f*_*p*_, a Fermi function $f_{p}\left(p\right)=\frac{1}{1+\exp\left(\frac{p_{\mathrm{cross}-p}}{p_{\mathrm{width}}}\right)}$ is assumed (*p*_cross_: crossover mode number; *p*_width_: crossover width). *ν*_*i*__,*j*_ describes the conformational crossover from Gaussian statistics at distances *n* ≡ |*i* – *j*| ≤ *N*_e_ to compressed behavior |*i* – *j*|^2ν^ at larger distances along the chain. Again, we describe this spatial crossover by a Fermi-type crossover at *N*_e_, with width *n*_width_ taken from the structural results obtained from SANS:^14^ $f_{n}\left(n\right)=\frac{1}{1+\exp(\left(N_{e}-n)/n_{\mathrm{width}}\right)}$ , where *N*_e_ and *n*_width_ describe the monomer number at the crossover distance and the width of the transition, respectively. Finally, the crossover function for the fractal exponent *ν*_*i*__,*j*_ becomes *ν*_*i*__,*j*_ (*n*) = ν_1_ + (*ν*_f_ – ν_1_)*f*_*n*_ (*n*), with ν_1_ = 1/2 and *ν*_f_ = 1/*d*_f_. *T*_f_(*p*) = [1 + exp­((*p* – *p*_min_))/*p*_width_ ]^−1^ is the crossover function between the different dynamic scaling regimes and $p_{\min}=\frac{N}{N_{e,0}}$ is the number of elementary loops.

The com diffusion of polymer rings evidence three dynamic regimes: an early time subdiffusive motion with *D*_ring_ ∼ *t* ^*α*^, which is followed by the theoretically established *D*_ring_ ∼ *t* ^3/4^ dynamics, and finally by Fickian diffusion.^13,23^ For the blends, the fit was not able to distinguish two different subdiffusive regimes. Staying with one subdiffusive regime, we determined the exponent α_diff_ and the crossover mean squared displacement (MSD): ⟨*r*_cross_^2^⟩.

Long entangled linear chains display Rouse dynamics at local scales followed by local reptation, originally introduced by DeGennes^24^ and further developed by Monkenbusch et al.^25^ The details are presented in the SI.

The synthesis of the linear and ring polymers (molecular weights: h-ring: 44 kg/mol; d-ring and d-linear: 40 kg/mol) was performed by anionic polymerization. The details of the synthesis, characterization, and purification are generally outlined elsewhere^26^ and described in detail in the SI. For the linear samples, we used the intermediate linear products before the ring closure reaction. The molecular weight characterization of the PEOs was carried out by size-exclusion chromatography (SEC) (for details, see SI). The results are listed in Table S1. The blends were achieved in dissolving the proper amounts of ring and linear chains in benzene and subsequent freeze-drying. In each case, a volume fraction of ϕ_R_ = 0.1 of the rings was hydrogenated, the remaining ring amount as well as the linear chains were deuterated. Correspondingly, the neat linear melt contained ϕ_L_ = 0.1 hydrogenated linear molecules in the deuterated matrix. The blend compositions are listed in Table S2 of the SI. As shown in the SI, such prepared blends displayed a maximum viscosity around ϕ_R_ = 0.5.

The neutron experiments were performed at 413 K on the NSE spectrometer IN15 at the ILL.^27,28^ We used neutron wavelengths of λ = 10 Å and 13.5 Å. The resolution was measured on a carbon black sample; the background was obtained from the fully deuterated linear 40 kg/mol melt. The explored *Q* range extended from *Q* = 0.04 Å^–1^ to *Q* = 0.13 Å^–1^ (see also SI).

Starting from the low ring volume fraction side, in Figure 1 we present the spectra obtained from the RL35 and RL50 blends. For comparison, in Figure 1a (RL35) the result from the neat melt is displayed as solid lines. As is evident for RL35 (and for RL10, see SI), the corresponding spectra very well agree with those from the neat linear melt – the ring dynamics appears to be enslaved by the linear host. In the SI we present both the spectra from the neat melt as well as those from RL10.

**Figure 1:**
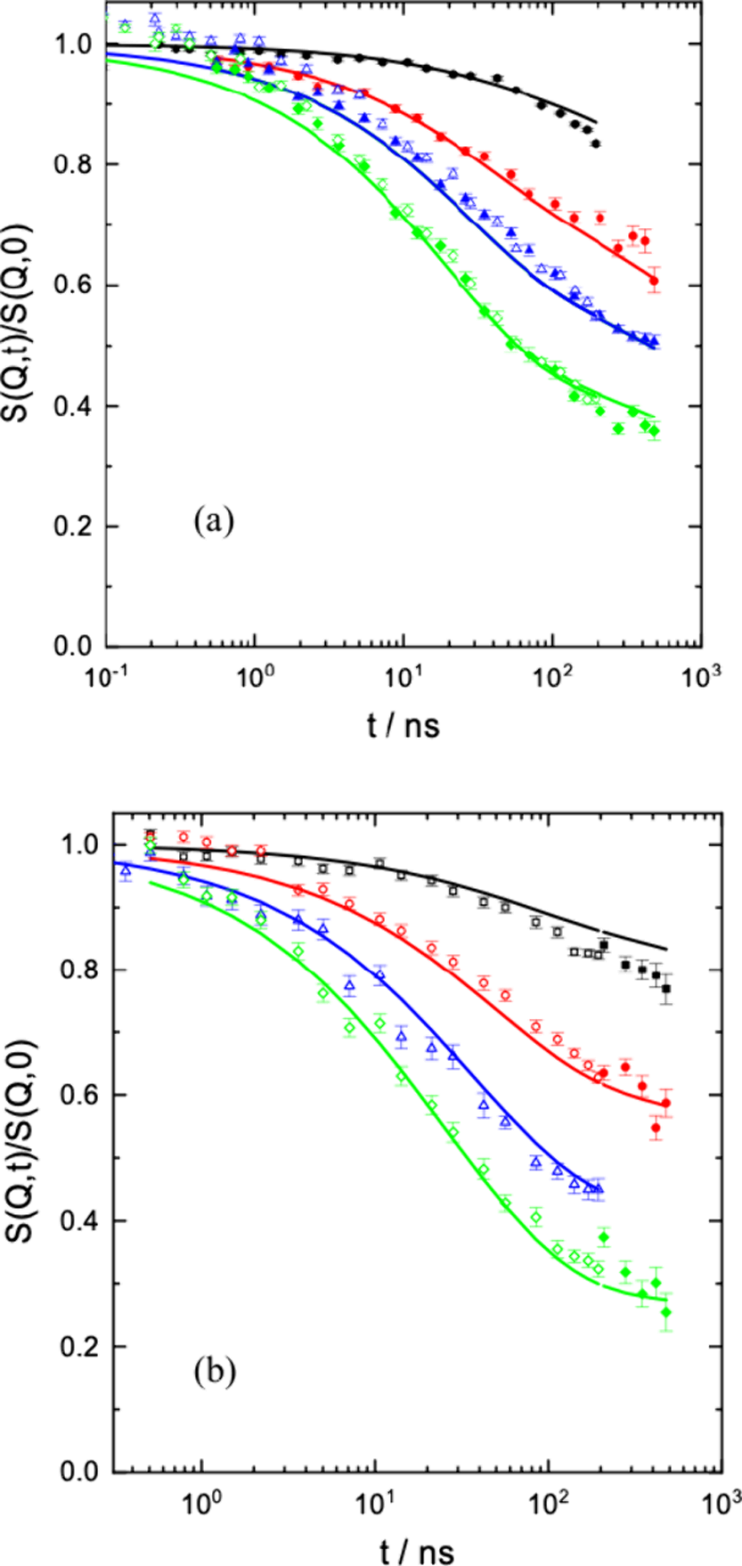
Comparison of the ring–linear spectra (a) from RL35 with the spectra from the linear melt (solid lines), as obtained from the joint fit of different linear molecular weights (see SI); (b) Fit of the RL50 spectra with the reptation model, including CLF varying α_*lik*_ and *N*_e_. The values of *Q* from above are 0.05, 0.08, 0.1, and 0.12 Å^–1^.

For RL50 slight but visible differences to the pure linear melt occur (compare, *e.g.*, data at *Q* = 0.12 Å^–1^). Fitting with the reptation model (see SI) including contour length fluctuation (CLF) yields a better representation of the data. In particular, the constraints mediated by the linear host and the CLF contribution (*α*_*lik*_) are reduced, and the apparent entanglement distance is increased from *N*_e_ = 69.5 to 96.6. The achieved fitting parameters are displayed in Table 1. We note that with varying only two parameters, the entanglement length *N*_e_ and α_*lik*_, very good fits can be obtained.

**Table 1:** Fit Parameters Achieved with Local Reptation Model for the Various Samples^a^

sample	*N*_e_	α_*lik*_	χ^2^
linear PEO/RL10/RL35	69.5 ± 0.3	2.44 ± 0.02	8.78
RL50	96.6 ± 2.2	0.81 ± 0.12	6.03

a *N*_e_: entanglement distance; α_*lik*_: CLF constant for neat melt data (see SI).

For RL50 slight but visible differences to the pure linear melt occur (compare, *e.g.*, data at *Q* = 0.12 Å^–1^). Fitting with the reptation model (see SI) including contour length fluctuation (CLF) yields a better representation of the data. In particular, the constraints mediated by the linear host and the CLF contribution (α_*lik*_) are reduced, and the apparent entanglement distance is increased from *N*_e_ = 69.5 to 96.6. The achieved fitting parameters are displayed in Table 1. We note that by varying only two parameters, the entanglement length *N*_e_ and α_*lik*_, very good fits can be obtained.

We also checked whether the observed change of the ring- to linear-type spectra might result from enhanced visibility of the linear chains due to their effective contrast in the blend as described by RPA.^29^ As shown in the SI, application of the RPA treatment does not explain the “plateau-like” retardation of the ring dynamics.

To model the ring rich spectra from RL95 and RL75 we needed an estimate for the Fickian diffusion coefficient of the ring. As shown in the SI, we estimated *D*_ring_ for these blends based on the zero-shear viscosity data. We start with RL95 and fix the Fickian diffusion coefficient to that estimated from viscosity measurements to $D_{\mathrm{Fick}}=0.019\frac{{Å}^{2}}{\mathrm{ns}}$ . As mentioned above, for the blend, we replaced the 2 crossovers by one and fitted both, ⟨*r*_cross_^2^⟩ and the corresponding subdiffusional exponent α_diff_. Figure 2a displays the result. We obtain an excellent fit with α_diff_ = 0.52 ± 0.007 and a crossover ⟨*r*_cross_^2^⟩ = 7600 Å^2^. The corresponding length $\sqrt{r_{\mathrm{cross}}^{2}}=87Å$ amounts to about half of the linear chain end-to-end distance *R*_e_ = 180 Å. In addition, the first ring mode (*p* = 2) is suppressed.

**Figure 2:**
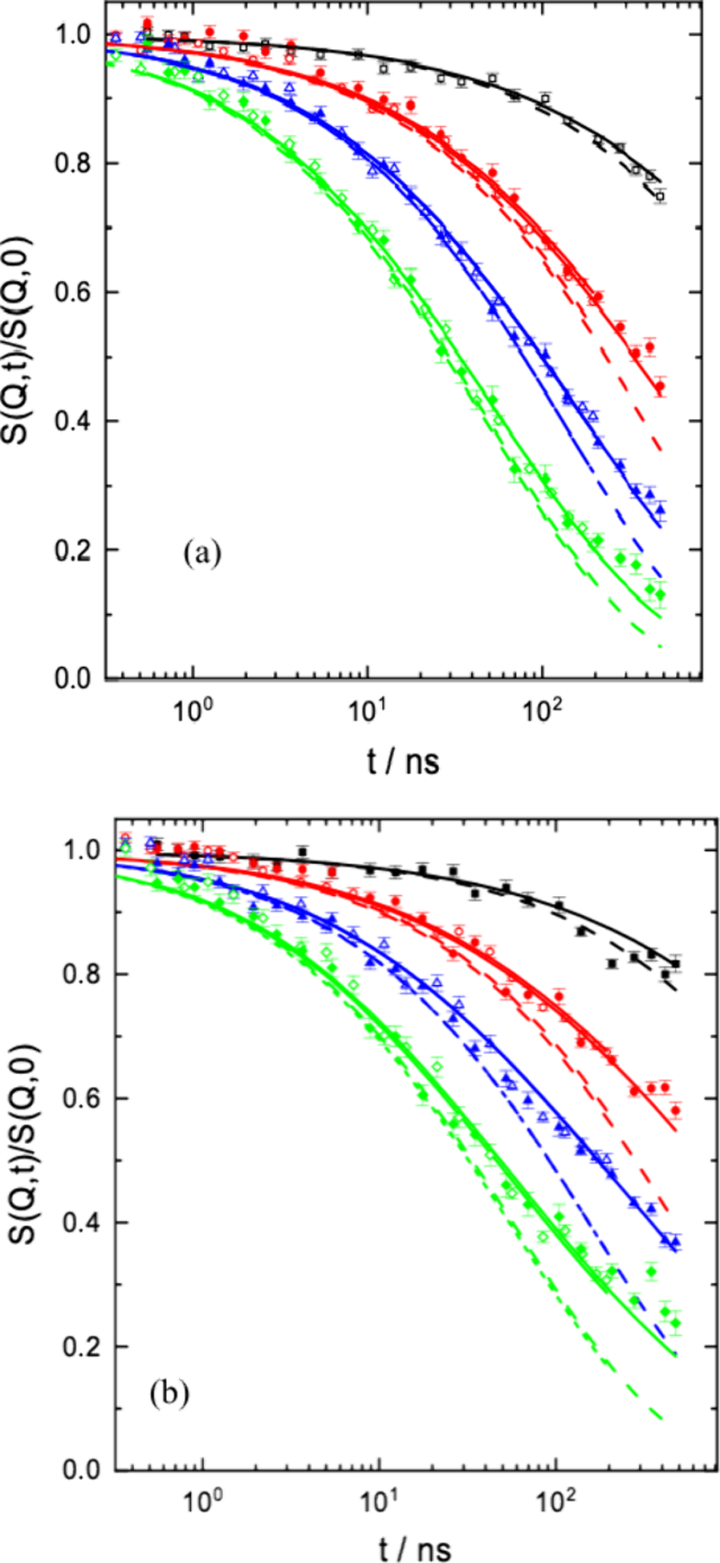
Comparison of the ring–linear spectra for RL95 (a) and RL75 (b), including mode analysis and fixing *D*_Fick_ to the values estimated from viscosity with (solid lines) and without (dashed lines) mode suppression. The values of *Q* from above 0.05, 0.08, 0.1, and 0.12 Å^–1^.

Now, we follow the same approach for the RL75 spectra, where the linear volume fraction amounts to 0.25. According to our estimates (SI), then the Fickian diffusion coefficient should be strongly reduced to $D_{\mathrm{Fick}}=0.0026\frac{{Å}^{2}}{\mathrm{ns}}$ , a value hardly visible in the NSE time window. Figure 2b (solid lines) shows the result. Again, a good fit is achieved; the crossover MSD becomes 32240 Å^2^, very close to *R*_e_^2^ = *l*_seg_^2^ *N* = 32260 Å^2^. In addition, now modes *p* = 2 and *p* = 4 are suppressed. To evidence the significance of the fitted mode suppression, Figure 2 compares the ring spectra with the achieved fits including mode suppression (solid lines) with a description without mode suppression (dashed lines). While the consideration of strongly reduced mode amplitudes leads to a very good description (solid lines) of the experimental spectra, the dashed lines show that including the full mode spectrum cannot describe the retarded spectral decay for times above 10 to 20 ns. Table 2 displays the fitting parameters achieved.

**Table 2:** Fit Parameters Achieved with the Ring Dynamic Structure Factor for the Various Samples^a^

sample	α_diff_	⟨*r*_cross_^2^⟩	*p*_min_	*p*_width_
RL95	0.520 ± 0.003	7600	2.09 ± 0.94	0.16 ± 1.78
RL75	0.50 ± 0.01	32240 ± 6900	3.6 ± 0.32	0.19 ± 0.53

a The diffusion coefficients were fixed to the values derived from viscosity measurements (see SI); the Rouse rate *Wl*4 = 14890 Å^4^/ns (PEO8K) and the ring fractal dimension ν_f_ = 0.45 were taken from ref 13.

Figure 3 summarizes our observations on symmetric ring-linear blends. The results from RL35 are not shown. RL35 cannot be distinguished from RL10. The spectra clearly show a dynamic crossover from ring-like to local reptation-like relaxation. Allowing for the reduction of the amplitudes of some large wavelength modes (RL95: mode *p* = 2; RL75: modes *p* = 2 and *p* = 4) the ring rich blends (RL95 and RL75) are well described by the ring dynamic structure factor including an estimated contribution from center of mass displacement. For linear reach blends, toward longer times the spectra bend away from the ring dynamics structure factor *S*_ring_ (*Q*,*t*) and show the typical behavior of a chain performing local reptation.^25^ As it appears, center of mass diffusion is virtually absent, and the dynamics is enslaved by that of the entangled linear melt.

**Figure 3:**
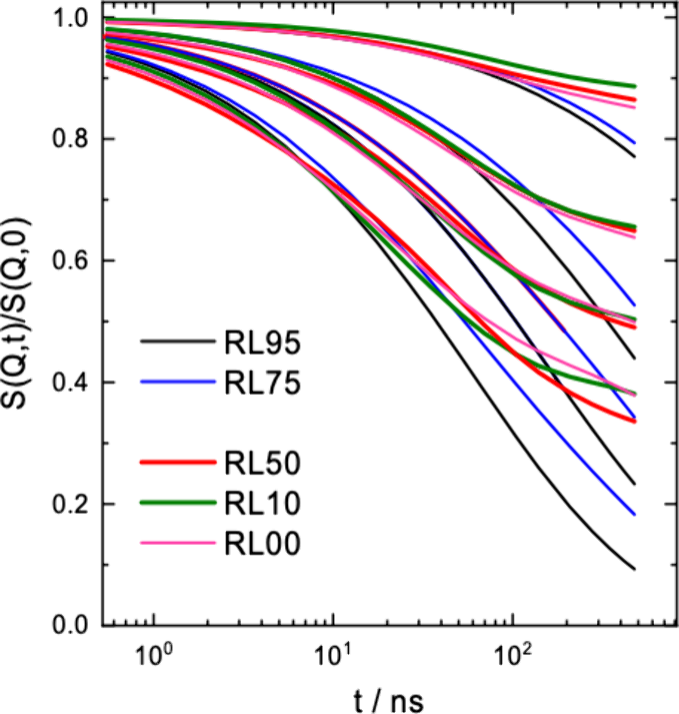
Comparison of the spectra from the blends with different ring volume fractions, from 0.1 to 0.95 (see legend). RL00 indicates pure linear melt. A clear transition from ring-like to linear-like dynamics is observed for the ring concentration ϕ_R_ ≈ 0.5.

In this work we have investigated the dynamics of PEO rings in a symmetric blend with linear PEO with *M*_w_ = 40 kg/mol over the full volume fraction range. The NSE spectra display a well pronounced crossover from ring-like dynamics at high ϕ_R_ to local reptation type dynamics at lower ϕ_R_ with a crossover ϕ_R_ around 50%. This crossover concurs in the same ring-linear concentration regime, where the maximum viscosity of the blend is observed. For higher linear concentration the ring dynamics is completely enslaved by the linear chains. Recent large-scale bead–spring MD simulations display a relative viscosity increase from both sides of the composition diagram with a maximum around ϕ_R_ ≅ 0.5.^7^ We note that for the much stiffer polystyrene (PS) the maximum viscosity is found for ϕ_R_ ≅ 0.4.^30^ At small ϕ_R_, rings are interwoven in the entanglement network of the linear chains and can only diffuse if they are released by all reptating linear chains that thread the rings.^15^ Experimentally, PFG-NMR measurements on various ring-linear blends also reveal that rings have a longer terminal relaxation time than that of the linear matrix.^16^

At low ring volume fraction, ϕ_R_ ≤ 0.3, we observe ring dynamics that is fully enslaved by the linear host. Thus, the relaxing ring sections lose their topological identity and are forced to move cooperatively with the linear majority – the constraints to motion are dictated by the entanglement network of the linear chains. We note that very recently we have demonstrated cooperative dynamics within the entanglement tube.^31,32^ The observation qualitatively agrees with the notion of rings “interwoven in the entanglement network”. Such a picture also supports our earlier observation of a large negative Flory–Huggins χ_FH_ parameter in ring linear blends favoring strong ring-linear interdigitation.^33^

In the intermediate concentration regime, the primitive-path analysis (PPA) of simulation results was interpreted by the fact that replacing linear chains by rings removes linear–linear entanglements and replaces them by topologically different constraints due to ring linear threading.^15^ For RL50 (ϕ_R_ = 0.5) we found the spectral shapes to be still close to the local reptation profiles, but the topological constraints enacted by the entanglement network of the host were found to be weakened (*N*_e_ ≅ 100 compared to *N*_e_ ≅ 70 for the neat linear melt). Our observations are correlating well with the findings of the PPA. It is interesting to note that at a point, where the effect of the entanglement network is weakened, the strongest enhancement of the viscosity is found.

When the fraction of linear chains becomes small, the entanglement network created by the linear chains must breakdown; the rings do not form persistent entanglements. We find that at low linear volume fraction the relaxation dynamics crosses over to ring like spectral shapes (ϕ_R_ = 0.75 and 0.95, see Figure 2). Nevertheless, the ring translational diffusion is strongly affected caused by ring-linear threading. A signature of the increasing number of threadings is also the augmented mode suppression in RL75. Concerning the internal ring relaxation, the first fully active mode for RL95 is *p* = 4, while for RL75 it is the *p* = 6 mode. Its relaxation time is a factor of 62/42 = 2.25 times shorter than the *p* = 4 mode. On the other hand, the full ring relaxation needs the constraint release by the linear chains which corresponds to a much longer time.

To what extend is our experiment able to inform also about terminal times that are far beyond the NSE time window (*t* ≅ 500 ns). In the simulations the diffusive relaxation times τ_d_ are computed as the average time required for a chain to diffuse a distance equal to 3*R*_g_ (*R*_g_: radius of gyration). For the linear chains the diffusional time changes very little with ϕ_R_. On the other hand, the ring relaxation time increases strongly with decreasing ϕ_R_, in the simulation by about 2 orders of magnitude.^7^ The NSE time frame does not reach the regime of Fickian diffusion. But based on our results for the complex diffusion properties including the estimated diffusion coefficient and the fitted subdiffusive dynamics, we can give a good estimation: For RL95 compared to RL100 (pure rings^13^) the time needed to reach 3*R*_g_ is prolonged from about 40 μs to about 115 μs i.e. a factor of 2.8: the ratio of the simulated terminal times τ_d_ yields, i.e., a factor of 2.5; for RL75 the diffusion time to reach 3*R*_g_ increases to about 300 μs, i.e., a factor of 7.5 compared to a simulated ratio of about 10.

According to the simulation results, at low values of ϕ_R_, the number of ring–linear threading amounts *N*_t_ ≅ 50, while for ϕ_R_ > 0.75*N*_t_ ≅ 1; even for ϕ_R_ → 1, each linear chain is still passing through several rings;^15^ and some rings will be double threaded by more than one linear chain. From our measurements we find that the characteristic MSDcom, a ring is undergoing before ring diffusion becomes Fickian, amounts to about 1/2*R*_e_^2^ for RL95 and *R*_e_^2^ for RL75, meaning that at ϕ_R_ = 0.75; the threaded rings on average have to move a distance spanned by the end-to-end length of the linear chain in the blend. Intuitively this makes sense; the ring has to lose all its initial threading before it may diffuse in an unconstrained way.

## Supplementary Material



## Notes

Characteristics of synthesized polymers and sample compositions; Consideration of dynamics of entangled linear chains in the melt; Description of the neutron data correction and viscosity experiment; Estimation of the ring Fickian diffusion coefficient based on viscosity data and random phase approximation (RPA) analysis (PDF)

## References

[1] Klein J. (1986). Dynamics of
entangled linear, branched, and cyclic polymers. Macromolecules.

[2] McKenna G. B., Plazek D. J. (1986). The viscosity of
blends of linear and cyclic molecules
of similar molecular mass. Polym. Commun..

[3] Mills P. J., Mayer J. W., Kramer E. J., Hadziioannou G., Lutz P., Strazielle C., Rempp P., Kovacs A. J. (1987). Diffusion
of polymer rings in linear polymer matrices. Macromolecules.

[4] Roovers J. (1988). Viscoelastic
properties of polybutadiene rings. Macromolecules.

[5] Roovers J., Toporowski P. M. (1988). Synthesis
and characterization of ring polybutadienes. J. Polym. Sci. B Polym. Phys..

[6] Kapnistos M., Lang M., Vlassopoulos D., Pyckhout-Hintzen W., Richter D., Cho D., Chang T., Rubinstein M. (2008). Unexpected
power-law stress relaxation of entangled ring polymers. Nat. Mater..

[7] Halverson J. D., Grest G. S., Grosberg A. Y., Kremer K. (2012). Rheology of Ring Polymer
Melts: From Linear Contaminants to Ring-Linear Blends. Phys. Rev. Lett..

[8] Gooßen S., Krutyeva M., Sharp M., Feoktystov A., Allgaier J., Pyckhout-Hintzen W., Wischnewski A., Richter D. (2015). Sensing Polymer Chain Dynamics through Ring Topology:
A Neutron Spin Echo Study. Phys. Rev. Lett..

[9] Ge T., Panyukov S., Rubinstein M. (2016). Self-Similar
Conformations and Dynamics
in Entangled Melts and Solutions of Nonconcatenated Ring Polymers. Macromolecules.

[10] Tsalikis D. G., Koukoulas T., Mavrantzas V. G., Pasquino R., Vlassopoulos D., Pyckhout-Hintzen W., Wischnewski A., Monkenbusch M., Richter D. (2017). Microscopic Structure, Conformation, and Dynamics of
Ring and Linear Poly­(ethylene oxide) Melts from Detailed Atomistic
Molecular Dynamics Simulations: Dependence on Chain Length and Direct
Comparison with Experimental Data. Macromolecules.

[11] Tsalikis D. G., Mavrantzas V. G. (2020). Size and
Diffusivity of Polymer Rings in Linear Polymer
Matrices: The Key Role of Threading Events. Macromolecules.

[12] Parisi D., Kaliva M., Costanzo S., Huang Q., Lutz P. J., Ahn J., Chang T., Rubinstein M., Vlassopoulos D. (2021). Nonlinear
rheometry of entangled polymeric rings and ring-linear blends. J. Rheol (N Y N Y).

[13] Kruteva M., Monkenbusch M., Allgaier J., Holderer O., Pasini S., Hoffmann I., Richter D. (2020). Self-Similar Dynamics of Large Polymer
Rings: A Neutron Spin Echo Study. Phys. Rev.
Lett..

[14] Kruteva M., Allgaier J., Monkenbusch M., Porcar L., Richter D. (2020). Self-Similar
Polymer Ring Conformations Based on Elementary Loops: A Direct Observation
by SANS. ACS Macro Lett..

[15] O’Connor T. C., Ge T., Grest G. S. (2022). Composite entanglement
topology and extensional rheology
of symmetric ring-linear polymer blends. J.
Rheol (N Y N Y).

[16] Kruteva M., Allgaier J., Richter D. (2017). Direct Observation
of Two Distinct
Diffusive Modes for Polymer Rings in Linear Polymer Matrices by Pulsed
Field Gradient (PFG) NMR. Macromolecules.

[17] Vigil D. L., Ge T., Rubinstein M., O’Connor T. C., Grest G. S. (2024). Measuring Topological
Constraint Relaxation in Ring-Linear Polymer Blends. Phys. Rev. Lett..

[18] Parisi D., Ahn J., Chang T., Vlassopoulos D., Rubinstein M. (2020). Stress Relaxation
in Symmetric Ring-Linear Polymer Blends at Low Ring Fractions. Macromolecules.

[19] Nam S., Leisen J., Breedveld V., Beckham H. W. (2009). Melt dynamics of
blended poly­(oxyethylene) chains and rings. Macromolecules.

[20] Doi Y., Takano A., Takahashi Y., Matsushita Y. (2022). Terminal relaxation
behavior of entangled linear polymers blended with ring and dumbbell-shaped
polymers in melts. Rheol. Acta.

[21] Crysup B., Shanbhag S. (2016). What Happens When Threading
is Suppressed in Blends
of Ring and Linear Polymers?. Polymers (Basel).

[22] Mo J., Wang J., Wang Z., Lu Y., An L. (2022). Size and Dynamics
of a Tracer Ring Polymer Embedded in a Linear Polymer Chain Melt Matrix. Macromolecules.

[23] Kruteva M., Allgaier J., Monkenbusch M., Hoffmann I., Richter D. (2021). Structure
and dynamics of large ring polymers. J. Rheol
(N Y N Y).

[24] De
Gennes P. G. (1981). Coherent scattering by one reptating chain. J. Phys. (Paris).

[25] Monkenbusch M., Kruteva M., Richter D. (2023). Dynamic structure factors of polymer
melts as observed by neutron spin echo: Direct comparison and reevaluation. J. Chem. Phys..

[26] Hövelmann C. H., Gooßen S., Allgaier J. (2017). Scale-Up Procedure for the Efficient
Synthesis of Highly Pure Cyclic Poly­(ethylene glycol). Macromolecules.

[27] Farago B., Falus P., Hoffmann I., Gradzielski M., Thomas F., Gomez C. (2015). The IN15 upgrade. Neutron News.

[28] Kruteva, M. ; Allgaier, J. ; Czakkel, O. ; Falus, P. ; Hoffmann, I. ; Monkenbusch, M. ; Richter, D. Exploring composite topological constraints in ring-linear blends. Institut Laue-Langevin (ILL), 2024, 10.5291/ILL-DATA.9-11-2149.

[29] Monkenbusch M., Kruteva M., Zamponi M., Willner L., Hoffman I., Farago B., Richter D. (2020). A practical method to account for
random phase approximation effects on the dynamic scattering of multi-component
polymer systems. J. Chem. Phys..

[30] Vlassopoulos, D. Viscosity of Ring-Linear Polystyrene Blends (Unpublished).

[31] Zamponi M., Kruteva M., Monkenbusch M., Willner L., Wischnewski A., Hoffmann I., Richter D. (2021). Cooperative
Chain Dynamics of Tracer
Chains in Highly Entangled Polyethylene Melts. Phys. Rev. Lett..

[32] Kruteva M., Zamponi M., Hoffmann I., Allgaier J., Monkenbusch M., Richter D. (2021). Non-Gaussian and Cooperative Dynamics of Entanglement
Strands in Polymer Melts. Macromolecules.

[33] Kruteva M., Monkenbusch M., Allgaier J., Pyckhout-Hintzen W., Porcar L., Richter D. (2023). Structure
of Polymer Rings in Linear
Matrices: SANS Investigation. Macromolecules.

