# Supplementary material for: Dynamics of Polymer Rings in Ring-Linear Blends by Neutron Spin Echo Spectroscopy
Source: ACS Macro Lett. 2025 Sep 15;14(10):1396–401. doi: 10.1021/acsmacrolett.5c00507 (PMC12548356; doi:10.1021/acsmacrolett.5c00507)
Supplement: Supplementary file 1 [file mz5c00507_si_001.pdf]

# DYNAMICS OF POLYMER RINGS IN RING-LINEAR BLENDS BY NEUTRON SPIN ECHO SPECTROSCOPY

## Supporting information

Margarita Kruteva,[1] Jürgen Allgaier,[1] Michael Monkenbusch,[1] Peter Falus ,[2] Katerina Peponaki,[3,4] Dimitris Vlassopoulos,[3,4] Dieter Richter[1]

[1]Jülich Center for Neutron Science, Forschungszentrum Jülich, 52428 Jülich, Germany
[2]Institut Laue-Langevin (ILL), 71 rue des Martyrs, 38042 Grenoble, Cedex 9, France
[3]FORTH, Institute for Electronic Structure and Laser, Heraklion 71110, Greece
[4]University of Crete, Department of Materials Science & Engineering, Heraklion 70013, Greece

## 1. Synthesized polymers and sample compositions:

The synthesis of the linear and ring PEOs is generally described elsewhere [1]. The hydrogenous 40 kg/mol PEO ring hR-PEO40k was obtained in a modified procedure by first synthesizing linear PEO using the potassium salt of di(ethylene glycol) monobenzyl ether (BnO–(EO)2–OK) as initiator for the polymerization of ethylene oxide (EO) and yielding monoalcoholic BnO–PEO–OH. This compound was immediately tosylated with a large excess of tosyl chloride (TosCl) to BnO–PEO–Tos, followed by the cleavage of the initial benzyl group with H2/PdC which leads to HO–PEO–OTos. This compound was used for the ring closure reactions. Unreacted linear precursor and chain coupled higher molecular weight linear byproducts were eliminated from the cyclization raw product by oxidizing the alcoholic chain ends to carboxylic acids and removing the oxidized material with the help of a basic ion exchange resin. Smaller quantities of higher molecular weight cyclic PEO were finally removed by fractionation using chloroform/heptane as solvent/nonsolvent pair. A more detailed description of this special strategy is given elsewhere [2]. The general procedure for the PEO ring synthesis is outlined in reference [1].
The deuterated 40 kg/mol PEO ring dR-PEO40k was synthesized in the same way as described above by replacing hydrogenous EO by deuterated ethylene oxide-d4 (dEO) (Cambridge Isotope Laboratories, deuteration degree 98 %).
For the linear samples, used in this work, dL-PEO40k, hL-PEO100k, and dL-PEO100k, the intermediate linear products before the ring closure reaction, BnO–PEO–OH, were used (see above). The synthesis of the linear 200k PEOs hL-PEO200k and dL-PEO200k is described in reference [3].
The molecular weight characterization of the PEGs was carried out by size-exclusion chromatography (SEC) using an Agilent 1260 Infinity SEC instrument equipped with a Wyatt DAWN Heleos II light scattering (LS) detector, an Optilab T-rex differential refractive index (RI) detector and with three PolyPore columns at 50 °C. The solvent was a mixture of THF, DMA, and acetic acid (84:15:1 by volume) at a flow rate of 1 mL/min.

**Table S1** Molecular weight characterization of the PEOs by SEC/LS. $M_n$ is the number average molecular weight, and the molecular weight distribution is given as $M_w/M_n$ whereby $M_w$ is the weight average molecular weight.

| | $M_n$ / kg/mol | $M_w/M_n$ |
|---|---|---|
| hR-PEO40k | 44.0 | 1.02 |
| dL-PEO40k | 41.5 | 1.01 |
| dR-PEO40k | 40.2 | 1.01 |
| hL-PEO100k | 97.6 | 1.01 |
| dL-PEO100k | 94.5 | 1.01 |
| hL-PEO200k | 190.0 | 1.04 |
| dL-PEO200k | 190.0 | 1.04 |

**Table S2** Blends of rings and linear PEO chains with $M_w = 40$ kg/mol and linear PEO chains as a reference that were investigated: $\phi_R$ ring volume fraction; $\phi_L$ linear volume fraction

| Blend | $\phi_R$ | $\phi_L$ | Laboratory labels |
|---|---|---|---|
| RL100 | 1.0 | 0.0 | RL-PEO40k-100 |
| RL95 | 0.95 | 0.05 | RL-PEO40k-95 |
| RL75 | 0.75 | 0.25 | RL-PEO40k-75 |
| RL50 | 0.5 | 0.5 | RL-PEO40k-50 |
| RL35 | 0.35 | 0.65 | RL-PEO40k-35 |
| RL10 | 0.1 | 0.9 | RL-PEO40k-10 |
| RL00 | 0.0 | 1.0 | RL-PEO40k-0 |
| L100 | 0.0 | 1.0 | L-PEO100k |
| PEO200 | 0.0 | 1.0 | L-PEO200k |

## 2. Dynamics of entangled linear chains in the melt

Long entangled linear chains display Rouse dynamics at local scales followed by local reptation, originally introduced by DeGennes [4] and further developed by Monkenbusch et al. [5]. Aside of refining the local reptation contribution, the Rouse dynamics within the tube was incorporated introducing a "Rouse blob" of size $R_e^2 = d^2$ describing the lateral Rouse motion within the tube of width "d". Following Guenza [6], also non-Gaussian chain dynamics, described in terms of a non-Gaussian parameter $\alpha(t)$, was considered. The scattering function for the Rouse-blob then becomes [5]:

$$S(Q,t) = \frac{1}{N_{blob}} \sum_{i,j}^{N_{blob}} exp\left[-\left(\frac{Q^2}{6}\right) f(Q^2) \langle [r_i(t) - r_j(0)]^2 \rangle\right] \quad \text{(S1)}$$

with

$$f(Q^2) = 1 - \frac{Q^2\alpha(t)\langle[r_m(t) - r_m(0)]^2\rangle}{12} + O(Q^4) \frac{\simeq}{Q \to 0} exp\left[-\frac{Q^2\alpha(t)\langle[r_m(t) - r_m(0)]^2\rangle}{12}\right] \qquad (S2)$$

and

$$\langle[r_m(t) - r_m(0)]^2\rangle = \frac{4N_{blob}l_{seg}^2}{\pi^2}\sum_{m,p}^{N_{blob}}\frac{1}{p^2}cos\left(\frac{p\pi m}{N_{blob}}\right)^2\left[1 - exp\left(-2W\left(1 - cos\left(\frac{p\pi}{N_{blob}}\right)\right)t\right]\qquad (S3)$$

Motivated by Guenza's simulation findings a logarithmic Gaussian function for the non-Gaussian parameter $\alpha(t) = \alpha_0 exp\left[-\left((\ln(t) - ln(t_{max}))/2\sigma^2\right)\right]$ has been introduced with $\alpha_{max} = \alpha_0$; $t_{max} = \tau_e$ $and$ $\sigma$ the width of the distribution .

Comparing the available spectra from linear PEO melts with different molecular weights, we realize that the PEO spectra exhibit a very significant dependence of the spectral shape on the chain length. Fig S1 displays the spectra from PEO linear melts with molecular weights of 200 kg/mol (PEO200); 100 kg/mol (L100); 40 kg/mol (RL00).

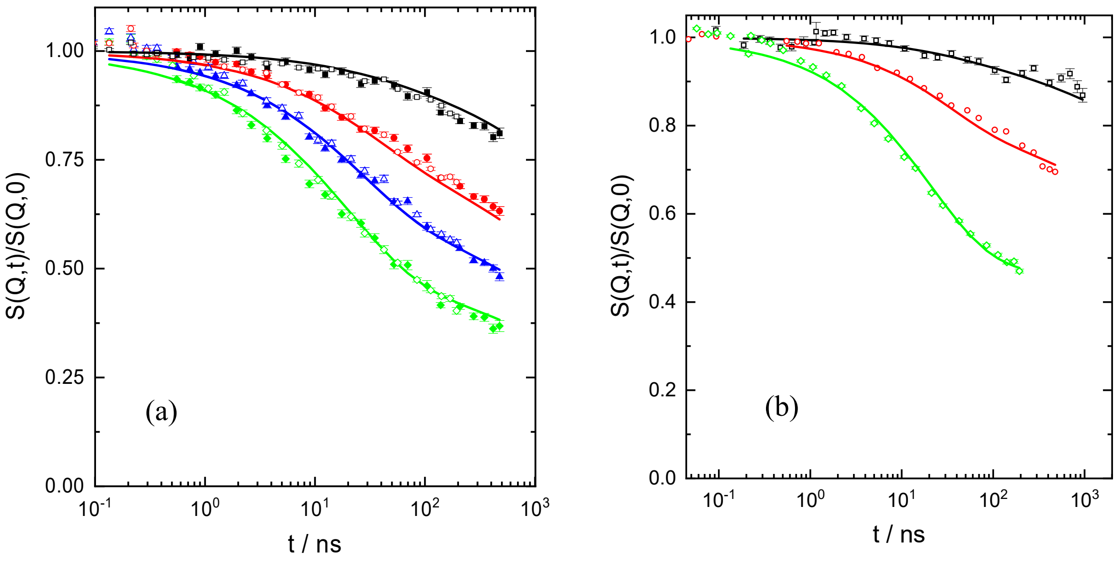

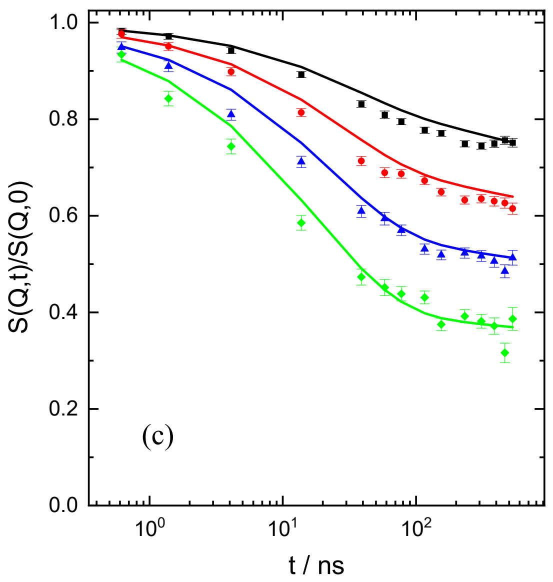


**Figure S1** Results of a joint fit of the spectra from the samples: (a): RL00 (40 kg/mol); (b): L100 (100 kg/mol); (c): PEO200 (200 kg/mol) with the reptation model including CLF (see text)

Such a difference of the spectra of different molecular weights in general relates to contour length fluctuations (CLF): by Rouse modes along the confining tube a chain will expand and retract towards the confinement, in this way a chain will "forget" about its initial tube and the effective confinement will be lifted from the ends. Based on Clarke and McLeish [7], Wischnewski et al. have calculated an explicit dynamic structure factor for a polymer undergoing reptation together with CLF [8].

$$S_{CLF}(Q,t) = 2\mu^2\left(2\mu + e^{-2\mu} + 2 - 4\mu s(t) - 4e^{-2\mu s(t)} + e^{-4\mu s(t)}\right) \qquad \text{(S4)}$$

With $\mu = Q^2 l^2/12$ and $s(t) = \frac{\alpha_{lik}}{Z}\frac{t}{\tau_e}$; $S_{CLF}(Q,t)$ corresponds to the creep term in Eq. 4. From simulations $\alpha_{lik} = 1.5$ was found. We note that for Z = 20 only a very small influence within the NSE window would be expected. As Fig. S1 shows for PEO this seems not to be the case. The RL00 and L100 spectra are grossly different and even between molecular weights of 100kg/mol and 200kg/mol differences are visible. Fitting with CLF included leads to a very good joint fit of the spectra from very different molecular weights (solid lines in Fig. S1). As shown in table 1 $\alpha_{lik} = 2.44 \pm 0.02$ evolves. To investigate the reason for the anomalous large contribution of CLF to the dynamic structure factor would be an interesting task, which, however, is beyond the aim of this manuscript. We further note that all spectra are well described by the same entanglement distance of $N_e = 69.5 \pm 0.3$.

The data for RL10 sample are presented in the Figure S2.

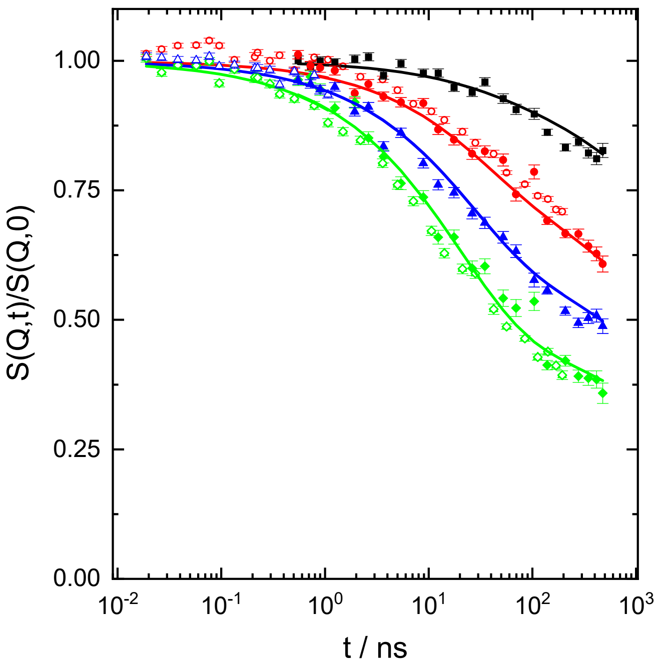


**Figure S2** Comparison of the ring-linear spectra from RL10 with the spectra from the linear melt (solid lines) as obtained from the joint fit of different linear molecular weights (see Fig.S1)

## 3. Data correction

We note that with exception of the measurement using $\lambda = 13.5$ Å at $Q = 0.05$ Å$^{-1}$ all spectra appeared to be properly corrected, in so far that all spectra for short times commence at 1.0. Systematically, the data from the low angle position corresponding to the center of the detector at $Q = 0.05$ Å$^{-1}$ and $\lambda = 13.5$ Å started about 3% to 5% above 1. We corrected these data by dividing the spectra by the overshoot that was obtained by averaging the first data points. After this procedure the corrected spectra well agreed with their counterparts measured with $\lambda = 10$ Å.

## 4. Viscosity of ring-linear poly(ethylene oxide) blends

Rheological measurements were performed on two rheometers: (i) a strain-controlled ARES (TA, USA) and an Anton Paar (Austria) MCR-702 double-head rheometer operating at strain-controlled mode. Temperature was controlled by a nitrogen convection oven in both cases. Stainless steel parallel plates of 8 mm diameter were used (specimen thickness of about 0.6 mm). The samples were loaded on the rheometer and shaped at 70°C (above the melting temperature), annealed and measured. Linear viscoelastic response, time-independence and reproducibility of the data were ensured by continuous tests. The terminal regime was probed in dynamic frequency sweep tests, and the dynamic viscosity was extracted, from which the zero-shear viscosity was determined. Some steady shear measurements were also performed and confirmed the viscosity values.

Impurities resulting from the synthesis are known to affect rheological measurements. This is particularly critical in the case of remaining unlinked linear chains and has been discussed in several

references [9–12]. In the present case, we cannot exclude the presence of unlinked linear chains but we note that based on the available rheological evidence (not presented here) the one-component ring does not exhibit a plateau modulus and, overall, the molar mass dependence of the zero-shear viscosity is consistent with that of critically purified rings and expectations from simulations and the FLG model [13–15].

The viscosity ratios (blend viscosity / ring viscosity: $\eta_B/\eta_R$) for the ring-linear PEO blends as a function of the ring volume fraction are displayed in Figure S4.

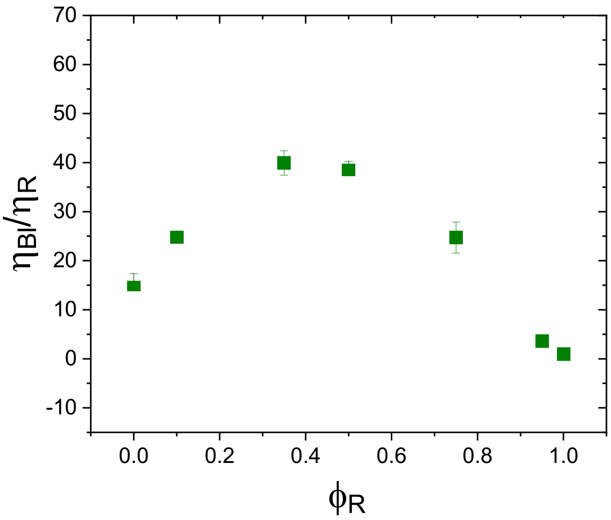


**Figure S4** Relative viscosities of ring linear blends of PEO 40 kg/mol rings in a blend with linear 40 kg/mol chains as a function of the ring concentration. The data are normalized to the viscosity of the neat ring melt.

## 5. Estimate of the ring Fickian diffusion coefficient based on viscosity data:

Unfortunately, there were no further samples available for proper PFG-NMR measurements. In the following we discuss how reasonable estimates for the Fickian diffusion coefficients of the rings within the blends may be obtained. For all blends the zero-shear viscosity has been measured. Also, for similar symmetric ring/linear blends simulation results are available that delivered both center of mass diffusion and zero shear viscosities. We show that based on the zero-shear viscosity reasonable estimates for the center of mass diffusion may be obtained

Halverson et al have performed simulations on ring linear blends and have evaluated both ring diffusion coefficients and zero shear viscosities. In a first step we proof that based on these simulation data $D_{ring} \sim 1/\eta_{blend}$ is well fulfilled.

**Table S3** Center of mass diffusion coefficients $D_{ring}$, zero shear viscosities $\eta_{blend}$ and the product of both for different ring volume fractions.

| $\phi_R$ | $D_{ring}$x$10^5$ | $\eta/10^2$ | $\eta_{blend}\, D_{ring}$ |
|---|---|---|---|
| 1.0 | 3.8 | 6.5 | 0.025 |

| | | | |
|---|---|---|---|
| 0.985 | 2.8 | 9.0 | 0.025 |
| 0.97 | 2.0 | 12 | 0.024 |
| 0.94 | 1.0 | 25 | 0.025 |
| 0.88 | 0.4 | 70 | 0.028 |
| 0.5 | 0.042 | 500 | 0.021 |

As it appears viscosity data may be well used to estimate the Fickian diffusion coefficients of the rings in the blends.

On the basis of the viscosity results displayed in chapter 4 we have estimated the ring diffusion coefficients: using the relation $D_{ring}(\phi_r) = D_{ring}(\phi_R = 1) * \eta_{blend}(\phi_R = 1)/\eta_{blend}(\phi_R)$ and the known $D_{ring} = 0.062 \left[\frac{Å^2}{ns}\right]$ at $\phi_L = 0$, we calculate the ring diffusion coefficients for the investigated blends.

| Blend | $\phi_L$ | $\eta_{blend}(\phi_L = 0)$ $/\eta_{blend}(\phi_L)$ | $D_{ring}\left[\frac{Å^2}{ns}\right]$ |
|---|---|---|---|
| RL100 | 0 | 1 | 0.062 |
| RL95 | 0.05 | 0.3 | 0.019 |
| RL75 | 0.25 | 0.042 | 0.0026 |
| RL50 | 0.5 | 0.026 | 0.0016 |

## 6. Role of RPA

Figure S5 shows the results of the application of the ring model with RPA influence of the linear chains in their reptation model compared to a sample with 20 percent rings [16]. It is clearly visible that the plateau like retardation of $S(Q,t)$ cannot be explained by the admixture of the linear chains signal due to RPA mediated visibility. Only at much lower ring content (in the order of 1 % or less) RPA effects become important.

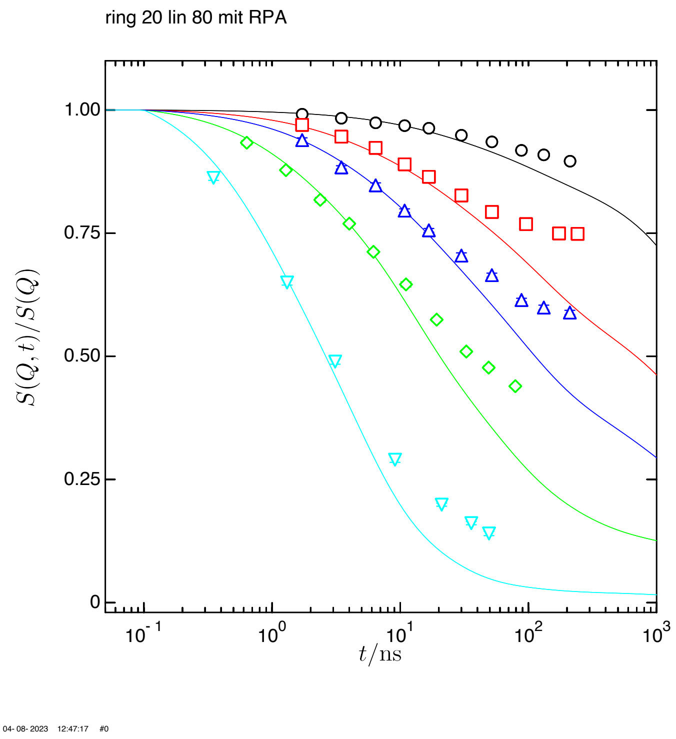


**Figure S5** Ring 20 kg/mol in linear PEO matrix 80 kg/mol. Lines reproduce the calculated spectra with the RPA contribution.

## References


[1] C. H. Hövelmann, S. Gooßen, and J. Allgaier, Scale-Up Procedure for the Efficient Synthesis of Highly Pure Cyclic Poly(ethylene glycol), Macromolecules **50**, 4169 (2017).

[2] M. Kruteva, J. Allgaier, M. Monkenbusch, I. Hoffmann, and D. Richter, Structure and dynamics of large ring polymers, J Rheol (N Y N Y) **65**, 713 (2021).

[3] B. J. Gold, W. Pyckhout-Hintzen, A. Wischnewski, A. Radulescu, M. Monkenbusch, J. Allgaier, I. Hoffmann, D. Parisi, D. Vlassopoulos, and D. Richter, Direct Assessment of Tube Dilation in Entangled Polymers, Phys Rev Lett **122**, 088001 (2019).

[4] P. G. De Gennes, Coherent scattering by one reptating chain, Journal de Physique **42**, 735 (1981).

[5] M. Monkenbusch, M. Kruteva, and D. Richter, Dynamic structure factors of polymer melts as observed by neutron spin echo: Direct comparison and reevaluation, J Chem Phys **159**, 034902 (2023).

[6] M. G. Guenza, Localization of chain dynamics in entangled polymer melts, Phys Rev E Stat Nonlin Soft Matter Phys **89**, 052603 (2014).

[7] N. Clarke and T. C. B. Mcleish, The Dynamic Structure Factor of a Star Polymer in a Concentrated Solution, Macromolecules **26**, 5264 (1993).

[8] A. Wischnewski, M. Monkenbusch, L. Willner, D. Richter, A. E. Likhtman, T. C. B. McLeish, and B. Farago, Molecular observation of contour-length fluctuations limiting topological confinement in polymer melts, Phys Rev Lett **88**, 583011 (2002).

[9] M. Kapnistos, M. Lang, D. Vlassopoulos, W. Pyckhout-Hintzen, D. Richter, D. Cho, T. Chang, and M. Rubinstein, Unexpected power-law stress relaxation of entangled ring polymers, Nat Mater **7**, 997 (2008).

[10] J. D. Halverson, G. S. Grest, A. Y. Grosberg, and K. Kremer, Rheology of Ring Polymer Melts: From Linear Contaminants to Ring-Linear Blends, Phys Rev Lett **108**, 038301 (2012).
[11] Y. Doi, A. Matsumoto, T. Inoue, T. Iwamoto, A. Takano, Y. Matsushita, Y. Takahashi, and H. Watanabe, Re-examination of terminal relaxation behavior of high-molecular-weight ring polystyrene melts, Rheol Acta **56**, 567 (2017).
[12] M. Kruteva, M. Monkenbusch, J. Allgaier, W. Pyckhout-Hintzen, L. Porcar, and D. Richter, Structure of Polymer Rings in Linear Matrices: SANS Investigation, Macromolecules **56**, 4835 (2023).
[13] K. Peponaki, D. G. Tsalikis, N. Patelis, G. Sakellariou, T. Chang, and D. Vlassopoulos, Revisiting the Viscosity of Moderately Entangled Ring Polymer Melts, Macromolecules **57**, 7263 (2024).
[14] R. Pasquino et al., Viscosity of ring polymer melts, ACS Macro Lett **2**, 874 (2013).
[15] A. Athanasiou et al., Unpublished data, (2025).
[16] S. Gooßen, M. Krutyeva, M. Sharp, A. Feoktystov, J. Allgaier, W. Pyckhout-Hintzen, A. Wischnewski, and D. Richter, Sensing Polymer Chain Dynamics through Ring Topology: A Neutron Spin Echo Study, Phys Rev Lett **115**, 148302 (2015).
